# Use of erbium laser in the treatment of persistent post-radiotherapy laryngeal edema: a case report and review of the literature

**DOI:** 10.1186/s12957-018-1480-9

**Published:** 2018-08-27

**Authors:** Aris I. Giotakis, Claus Pototschnig

**Affiliations:** Department of Otorhinolaryngology, Medical University of Innsbruck, Anichstrasse 35, 6020 Innsbruck, Austria

**Keywords:** Erbium, Laser microsurgery, Post-radiotherapy, Laryngeal, Edema

## Abstract

**Background:**

Post-radiotherapy laryngeal edema may affect the patients’ quality of life, leading to repeated treatment attempts, which include massage/physical therapy, inhalations, and/or tracheostomy.

**Case presentation:**

We report the surgical treatment approach of a 69-year-old patient with severe persistent post-radiotherapy laryngeal edema. After multiple inpatient admissions and failed conservative therapy, we used the erbium laser to treat the arytenoid edema. After repeated procedures, no complications were observed. The patient remained free of symptoms after 30 months of follow-up.

**Conclusions:**

The authors provide an easy-to-perform, safe, and quick surgical technique without non-severe or severe complications. Using this technique repeatedly, complications from excessive thermal damage with CO_2_ laser or unpleasant solutions such as tracheostomy can be avoided.

## Background

Post-radiotherapy laryngeal edema may result in hoarseness, airway compromise, and dysphagia. It may also affect the long-term patients’ quality of life, leading to repeated treatment attempts, which include massage/physical therapy, inhalations, and/or tracheostomy. In order to provide alternative methods to confront this condition, we report the use of erbium laser in the treatment of post-radiotherapy laryngeal edema of a 69-year-old male patient.

## Case presentation

**Fig. 1:**
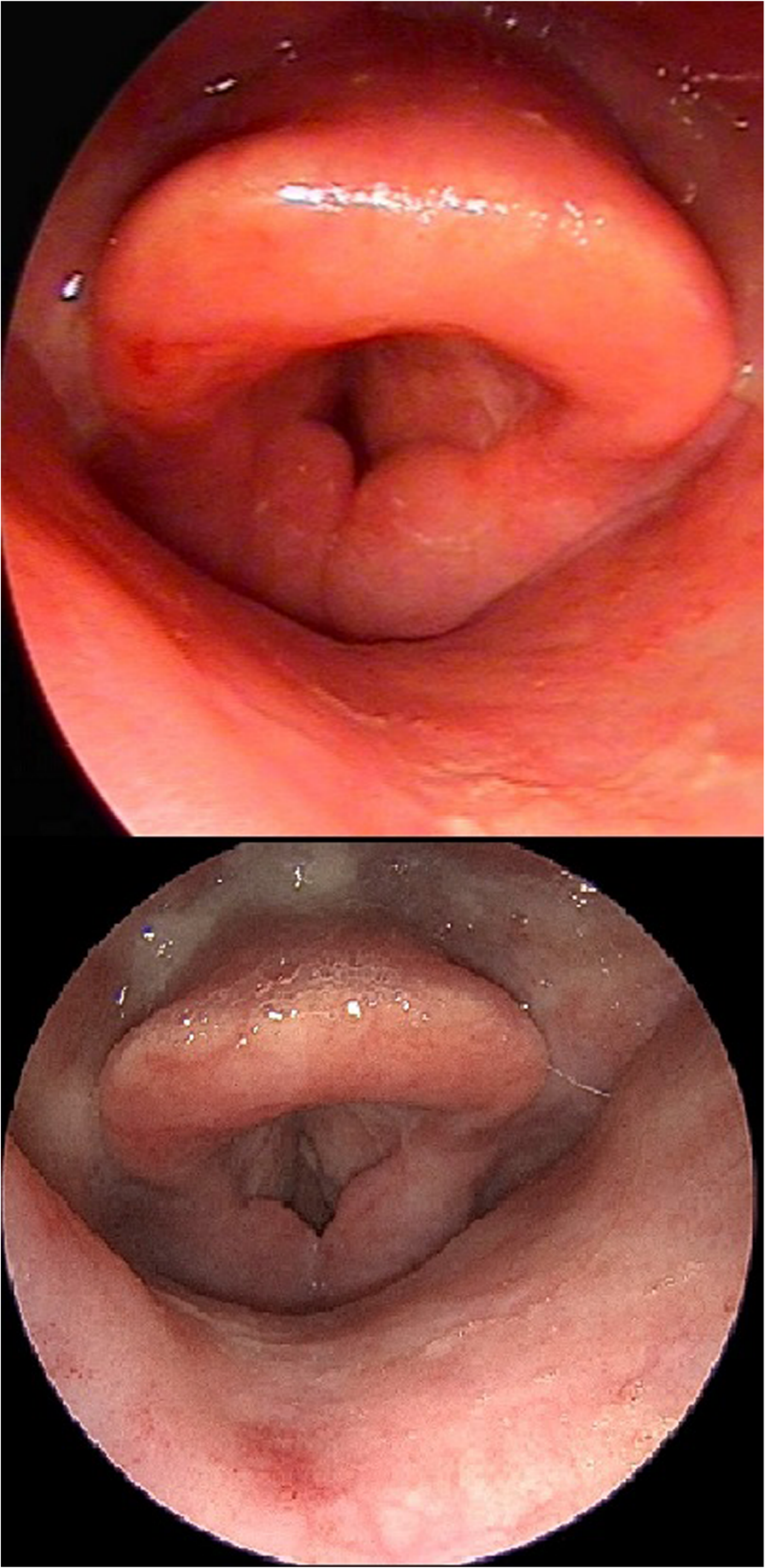
Upper: pictures before any interventions. Massive bilateral similar arytenoid edema (in respiratory position) causing dyspnea. Bottom: 30 months after the last surgery with erbium laser (in same respiratory position as preoperatively). No contact between anterior parts of the arytenoid area (in comparison to preoperatively) after treatment of both arytenoid areas with the erbium laser. Obvious difference in the edema of the anterior-posterior axis of the arytenoid area in comparison to preoperatively

We report a case of a 69-year-old male patient with post-radiotherapy laryngeal edema. The patient was treated with tumor resection, right selective neck dissection of levels II to IV, and adjuvant radiotherapy due to a pT_2_N_1_M_0_R_0_ oropharyngeal squamous cell carcinoma of the right tonsil. In the 2 years following radiotherapy, the patient was treated six times as an inpatient due to acute dyspnea. The endoscopic findings of the larynx always revealed a massive edema of the arytenoid area (Fig. 1, upper). Treatment included corticosteroid/adrenalin inhalation with systemic corticosteroids. Each time, subjective and objective recovery were transient. The endoscopic and radiologic findings revealed no indications of tumor recurrence. As an outpatient, the patient underwent multiple sessions of lymphatic massage drainage without improvement. Treatment with proton pump inhibitors also showed neither subjective nor objective benefits.

**Fig. 2:**
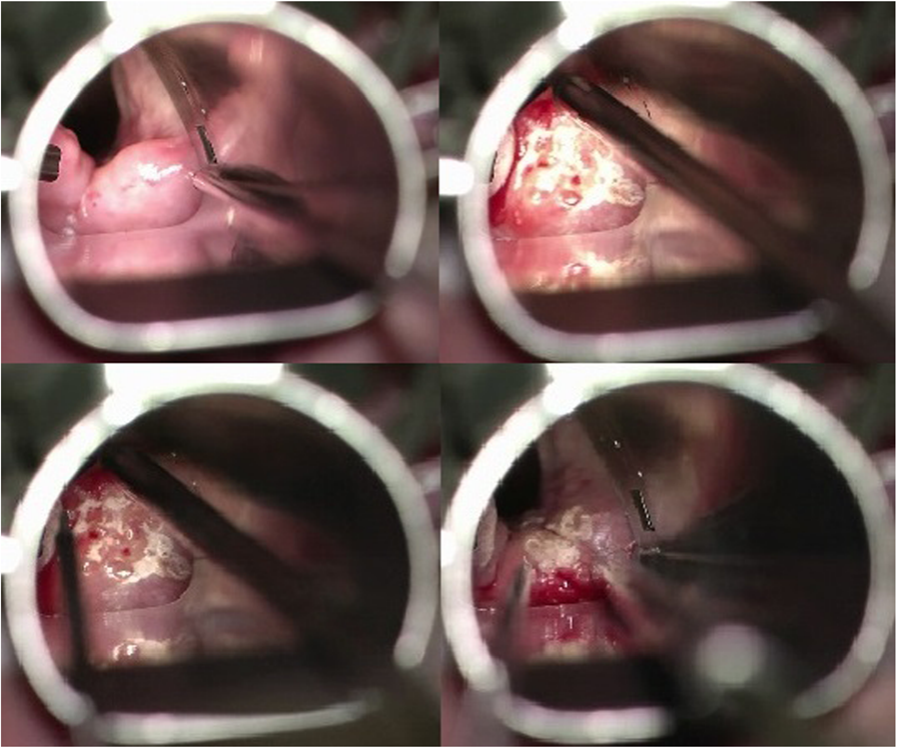
Pictures during surgery of the right arytenoid area with an erbium laser. Left upper: before the initiation of the procedure. The position of the microlaryngoscopy tube exposes the right arytenoid area; right upper: during “bombardment” of the cranial surface of the right arytenoid area with erbium laser to achieve a shrinking effect; left bottom: post-treatment picture. “Bombardment” of the cranial surface of the right arytenoid results in the production of white areas. The red points represent the holes created to empty the edema fluids; right bottom: after releasing the tension

Two and a half years after radiotherapy, the patient underwent transoral laser microsurgery of the arytenoid area. An erbium laser was used. The laser was set at 103 J/cm^2^ and 10 Hz. To prevent postoperative synechia and/or webs, only the right arytenoid was assessed. This intervention aimed to minimize the edema without causing severe thermal tissue damage, which could lead to additional edema. Therefore, the cranial surface of the right arytenoid was pulse targeted to achieve a shrinking effect. Subsequently, multiple targeted holes were made in the tissue. Edema fluid was emptied from the channels. The intraoperative effect was slightly obvious (Fig. 2). The patient remained under general anesthesia. The day after the procedure, microlaryngoscopy was performed. No additional edema was observed. The right arytenoid was still shrunken, and the patient was extubated.

**Fig. 3:**
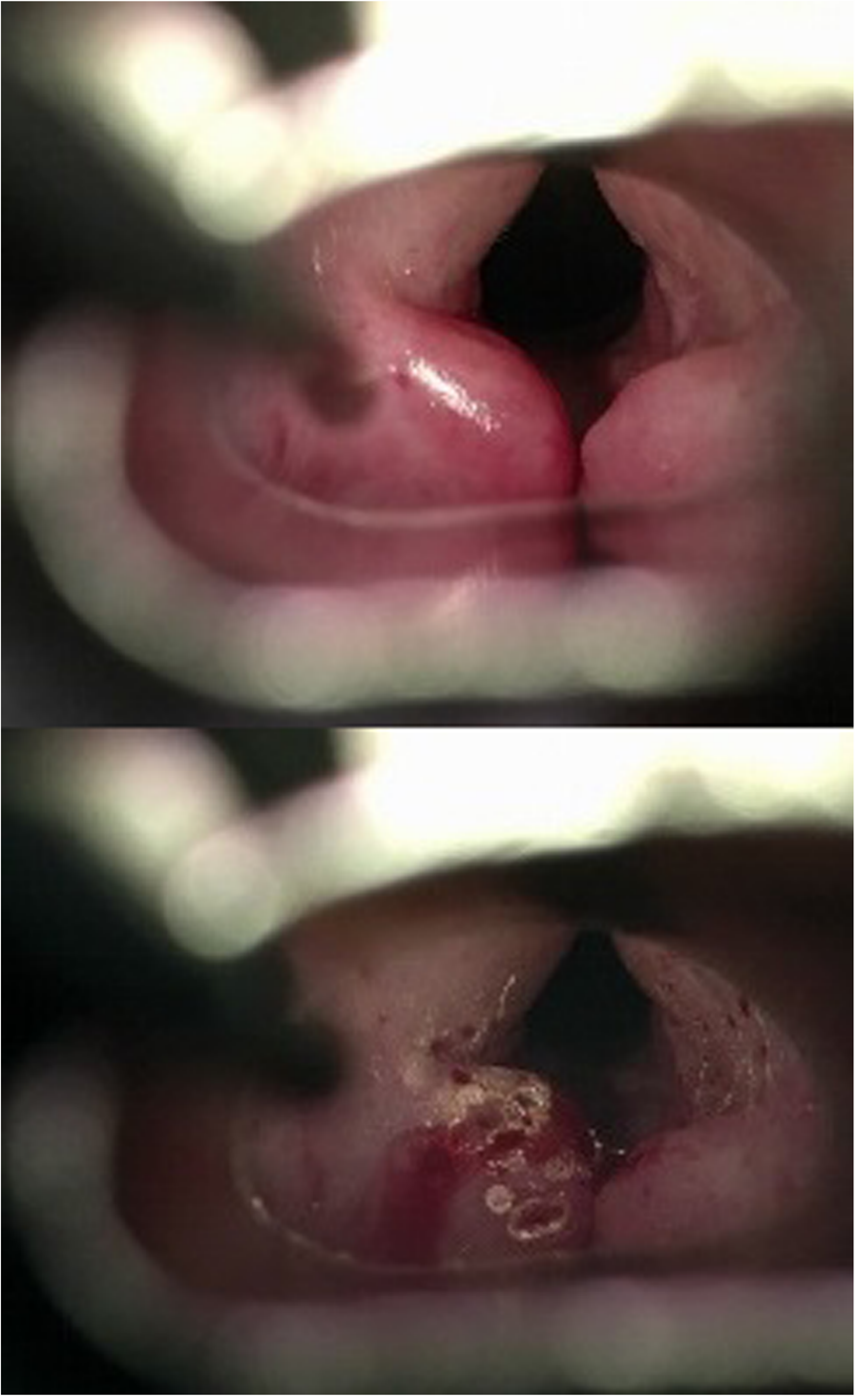
Pictures during surgery of the left arytenoid area with an erbium laser. Upper: before the initiation of the procedure. Obvious difference in the edema of the left non-treated swollen arytenoid in comparison to the already treated right arytenoid area. The right arytenoid area is healed after surgery 3 weeks ago. No scarring, synechiae, or webs are observed; Bottom: post-treatment picture. “Bombardment” of the cranial surface of the right arytenoid results in white areas. The red points represent the holes created to empty the edema fluids

The patient was admitted 2 weeks later to our department due to acute dyspnea. However, endoscopic examination of the larynx revealed a slight edema reduction of the right arytenoid. After conservative treatment with inhalation and systemic corticosteroids, the same procedure was performed for the left arytenoid, resulting in similar intraoperative edema reduction (Fig. 3). In January 2015, 2 months later, endoscopic findings revealed a slight edema reduction of the left arytenoid area, whereas the postoperative status of the right arytenoid remained stable. No synechiae, webs, or local swelling were observed. The patient also noted a slight improvement of the dyspnea. Thus, a new procedure was performed using the same surgical technique, this time in both arytenoids, with the same intraoperative findings. Again, no synechiae or webs were observed postoperatively.

During the next 6 months, the patient underwent three procedures in both arytenoids using the same surgical technique, with the same intraoperative findings. The erbium laser settings varied between 100 and 200 J/cm^2^ and 3 and 10 Hz, depending on the precise exposure of the cranial surface of the arytenoid area. No complications were observed. Endoscopic findings of the larynx at 2 months after the final procedure revealed a massive improvement. The patient experienced no symptoms. Thirty months after the final procedure, no additional edema was observed (Fig. 1, bottom).

## Discussion and conclusions

The erbium:yttrium-aluminum-garnet (erbium:YAG) laser has been in medical use for decades. While its clinical application in dermatology and dentistry is well documented [1–4], its use in laryngeal surgery has not been described. The authors of the current article performed a search of the data in the PubMed database using all possible combinations of the following keywords: “erbium,” “erbium:YAG,” “er:YAG,” “laryngeal,” “larynx,” and “arytenoid.” Five of the 8 publications retrieved were considered irrelevant. These publications concerned the use of erbium lasers and snoring, the use of a picosecond infrared laser in laryngeal tissue, the susceptibility of the laryngeal airway mask, laser reshaping of the costal cartilage transplantation, and the influence of erbium lasers in the fenestration of the inner ear. The three remaining publications were considered relevant. Excluding the German article, two of these studies compared the thermal damage of carbon dioxide (CO_2_) and erbium:YAG lasers in non-living animal and human laryngeal tissue [5–7].

The first study by Herdman and coauthors, conducted in 1993, examined the human vocal fold in vitro [5]. In this study, a continuous mode was used for the CO_2_ laser. The authors reported that charring is eliminated when using the erbium:YAG laser. Specifically, the depth of coagulated necrosis near the incision was reduced from 510 μm (± 75) with the CO_2_ laser to 23 μm (± 12) with the erbium:YAG laser. At the base of the incision, the same values were 125 μm (± 45) with the CO_2_ laser and 12 μm (± 8) with the erbium:YAG laser. The second study by Böttcher and coauthors, performed in 1994, used ex vivo porcine vocal folds [7]. In this study, the super-pulse mode was used for the CO_2_ laser. With the super-pulse mode, less thermal damage should occur compared to the continuous mode [8]. The authors reported that erbium:YAG incisions produced significantly decreased epithelial (236.44 μm) and subepithelial (72.91 μm) damage zones (*p* < 0.001) compared to the CO_2_ laser. Cutting gaps were significantly narrowed when using the CO_2_ laser (878.72 μm) compared to the erbium:YAG laser (1090.78 μm; *p* < 0.027). Collagen fibers along the erbium:YAG laser cutting edges were intact, without obvious carbonization, in contrast to the diffuse carbonization and tissue melting observed along the CO_2_ laser cutting edges. Both studies concluded that the erbium:YAG laser produces less thermal damage than the CO_2_ laser when used on the laryngeal tissue.

This physical ability originates from the ability of the erbium:YAG laser to emit light at 2.94 μm, which corresponds to the peak absorption of water for non-ionizing wavelengths. This absorption is approximately ten times greater than the absorption that occurs at a wavelength of 10.6 μm, which is emitted by the CO_2_ laser [7]. Therefore, in hydrated tissue, photon absorption from the erbium:YAG laser occurs over a shorter distance than that of a CO_2_ laser. Another study reported that the mean surface temperature increased only approximately 19 °C during the ablation of porcine skin with an erbium:YAG laser [8]. Both studies concluded that the thermal spread and temperature increases are limited when using an erbium:YAG laser.

Post-radiotherapy laryngeal edema occurs in 5 to 50% of patients treated with 45 and 80 Gy doses of radiotherapy, respectively, in the head and neck region [9]. Fu and coauthors reported that post-radiotherapy laryngeal edema without persistent or recurrent disease developed in 21/247 (8.5%) patients irradiated due to carcinoma of the vocal cord [10]. The addition of concurrent chemotherapy can double the risk of laryngeal edema [11]. Post-radiotherapy laryngeal edema can be treated conservatively and/or surgically. Conservative management includes adrenalin-corticosteroid inhalation, systemic corticosteroids, and/or massage/physical therapy. Surgical management includes tracheostomy or, in selected cases, functional laryngectomy, if aspiration is present. Recently, Lee and coauthors provided an alternative surgical approach to treat post-radiotherapy laryngeal edema [12]. Specifically, the authors reported four such cases treated with arytenoid resection with a CO_2_ laser. Airway widening was more than sufficient in all four cases and remained stable in follow-up evaluations. No permanent complications were observed. However, one patient experienced temporary vocal fold fixation for 6 months. The authors presumed that mucosal inflammation from the CO_2_ laser injury extending to the cricoarytenoid joint caused the fixation. They concluded that excessive CO_2_ laser injury to the cartilage can cause mucosal stenosis, stricture, and perichondritis. Crumley also reported that excessive laser injury to the arytenoid cartilage could cause cricoarytenoid joint injury with subsequent fusion of the arytenoid to the underlying cricoid [13].

In this case report, we describe the clinical use of an erbium laser (2.94 μm) for the treatment of post-radiotherapy laryngeal/arytenoid edema. Using an erbium laser, the depth of the coagulated necrosis and thermal damage should be sufficiently reduced compared to a CO_2_ laser. This technique is easy to perform. The erbium laser should be set between 100 and 200 J/cm^2^ and 3 and 10 Hz. Using the laser beam, the cranial surface of the arytenoid area is pulse targeted to achieve a shrinking effect. Then, multiple holes to empty the edema fluid are made in the cranial surface of the arytenoid. No resection or deep maneuvers adjacent to the cricoarytenoid joint are required. This technique can be quickly performed. An experienced surgeon can treat both arytenoids in approximately 15 min. No complications, synechiae, or webs were observed, and this technique can be considered safe. We performed this technique five times per arytenoid over 6 months. This method could be applied for severe cases. Airway widening was stable 30 months after the final surgery, and the patient experienced no symptoms.

In conclusion, we report the clinical use of an erbium laser to treat severe post-radiotherapy laryngeal edema. We provide an easy-to-perform, safe, and quick surgical technique without non-severe or severe complications. Using this technique repeatedly, complications from excessive thermal damage with a CO_2_ laser or unpleasant solutions, such as tracheostomy, can be avoided. Further studies are required to verify the scientific merit of this technique.

## Abbreviations

CO_2_: Carbon dioxide
Erbium:YAG: Erbium:yttrium-aluminum-garnet

## References

[1] Bertrand MF, Semez G, Leforestier E, Muller-Bolla M, Nammour S, Rocca JP (2006). Er:YAG laser cavity preparation and composite resin bonding with a single-component adhesive system: relationship between shear bond strength and microleakage. Lasers Surg Med.

[2] Takamori K, Furukawa H, Morikawa Y, Katayama T, Watanabe S (2003). Basic study on vibrations during tooth preparations caused by high-speed drilling and Er:YAG laser irradiation. Lasers Surg Med.

[3] Greene D, Egbert BM, Utley DS, Koch RJ (2000). In vivo model of histologic changes after treatment with the superpulsed CO (2) laser, erbium:YAG laser, and blended lasers: a 4- to 6-month prospective histologic and clinical study. Lasers Surg Med.

[4] Adrian RM (1999). Pulsed carbon dioxide and long pulse 10-ms erbium-YAG laser resurfacing: a comparative clinical and histologic study. J Cutan Laser Ther.

[5] Herdman RC, Charlton A, Hinton AE, Freemont AJ (1993). An in vitro comparison of the erbium: YAG laser and the carbon dioxide laser in laryngeal surgery. J Laryngol Otol.

[6] Luerssen K, Lubatschowski H, Ptok M (2007). Erbium:YAG laser surgery on vocal fold tissue. HNO.

[7] Bottcher A, Jowett N, Kucher S, Reimer R, Schumacher U, Knecht R (2014). Use of a microsecond Er:YAG laser in laryngeal surgery reduces collateral thermal injury in comparison to superpulsed CO2 laser. Eur Arch Otorhinolaryngol.

[8] Hobbs ER, Bailin PL, Wheeland RG, Ratz JL (1987). Superpulsed lasers: minimizing thermal damage with short duration, high irradiance pulses. J Dermatol Surg Oncol.

[9] Emami B, Lyman J, Brown A, Coia L, Goitein M, Munzenrider JE (1991). Tolerance of normal tissue to therapeutic irradiation. Int J Radiat Oncol Biol Phys.

[10] Fu KK, Woodhouse RJ, Quivey JM, Phillips TL, Dedo HH (1982). The significance of laryngeal edema following radiotherapy of carcinoma of the vocal cord. Cancer.

[11] Rancati T, Schwarz M, Allen AM, Feng F, Popovtzer A, Mittal B (2010). Radiation dose-volume effects in the larynx and pharynx. Int J Radiat Oncol Biol Phys.

[12] Lee HS, Kim SW, Kim WS, Lee KD (2010). Transoral CO (2) laser resection for post-radiation arytenoid edema. Clin Exp Otorhinolaryngol.

[13] Crumley RL (1993). Endoscopic laser medial arytenoidectomy for airway management in bilateral laryngeal paralysis. Ann Otol Rhinol Laryngol.

